# The VOICE study – A before and after study of a dementia communication skills training course

**DOI:** 10.1371/journal.pone.0198567

**Published:** 2018-06-11

**Authors:** Rebecca O’Brien, Sarah. E. Goldberg, Alison Pilnick, Suzanne Beeke, Justine Schneider, Kate Sartain, Louise Thomson, Megan Murray, Bryn Baxendale, Rowan H. Harwood

**Affiliations:** 1 School of Health Sciences, University of Nottingham, Nottingham, United Kingdom; 2 School of Sociology and Social Policy, University of Nottingham, Nottingham, United Kingdom; 3 Division of Psychology and Language Sciences, UCL, London, United Kingdom; 4 Dementia and Frail Older Persons PPI group, University of Nottingham, Nottingham, United Kingdom; 5 Division of Psychiatry and Applied Psychology, University of Nottingham, Nottingham, United Kingdom; 6 Simulated Patients Workshop Team (SPWT), Market Harborough, Leicestershire, United Kingdom; 7 Trent Simulation Centre, Nottingham University Hospitals NHS Trust, Nottingham, United Kingdom; 8 Department of Healthcare of the Older Person, Nottingham University Hospitals NHS Trust, Nottingham, United Kingdom; Taipei Veterans General Hospital, TAIWAN

## Abstract

**Background:**

A quarter of acute hospital beds are occupied by persons living with dementia, many of whom have communication problems. Healthcare professionals lack confidence in dementia communication skills, but there are no evidence-based communication skills training approaches appropriate for professionals working in this context. We aimed to develop and pilot a dementia communication skills training course that was acceptable and useful to healthcare professionals, hospital patients and their relatives.

**Methods:**

The course was developed using conversation analytic findings from video recordings of healthcare professionals talking to patients living with dementia in the acute hospital, together with systematic review evidence of dementia communication skills training and taking account of expert and service-user opinion. The two-day course was based on experiential learning theory, and included simulation and video workshops, reflective diaries and didactic teaching. Actors were trained to portray patients living with dementia for the simulation exercises. Six courses were run between January and May 2017. 44/45 healthcare professionals attended both days of the course. Evaluation entailed: questionnaires on confidence in dementia communication; a dementia communication knowledge test; and participants’ satisfaction. Video-recorded, simulated assessments were used to measure changes in communication behaviour.

**Results:**

Healthcare professionals increased their knowledge of dementia communication (mean improvement 1.5/10; 95% confidence interval 1.0–2.0; p<0.001). Confidence in dementia communication also increased (mean improvement 5.5/45; 95% confidence interval 4.1–6.9; p<0.001) and the course was well-received. One month later participants reported using the skills learned in clinical practice. Blind-ratings of simulated patient encounters demonstrated behaviour change in taught communication behaviours to close an encounter, consistent with the training, but not in requesting behaviours.

**Conclusion:**

We have developed an innovative, evidence-based dementia communication skills training course which healthcare professionals found useful and after which they demonstrated improved dementia communication knowledge, confidence and behaviour.

## Introduction

Across the world, people living with dementia (PLwD) are more likely to be admitted to hospital than those without dementia [1]. In the UK, a quarter of hospital beds are occupied by a person with dementia [2, 3]. PLwD dementia have problems with memory, understanding and communication [4]. Communication impairments can include difficulties with word finding, repetition of thoughts, lack of coherence in speech and difficulty understanding the language of others. These impairments can progress to a state where there is no intelligible speech [5]. Communication impairment is likely to be exacerbated by admission to hospital because of the unfamiliarity of people, place and activity within a busy, noisy and distracting environment.

### Communication in hospital

Much communication in hospital is around ‘tasks’ [6], for example asking a patient to take medication, washing and dressing, taking blood pressure, doing an assessment or physical examination. These tasks require active or passive co-operation from the patient. The communication needed to achieve this cooperation generally involves a sequence of interactions, such as: the healthcare professional (HCP) introducing themselves and the purpose of the task, obtaining agreement, gathering necessary information, supporting the patient to do a task, or doing a task for/to the patient and ending the encounter [7]. It can be important for the health and wellbeing of the patient that such tasks are completed, but co-operation is not always forthcoming. This is particularly the case when communication or cognitive impairments mean the patient with dementia does not understand what is being requested. Most acute hospital staff report having received little or no dementia-specific communication skills training [8], and HCPs report that they lack confidence in caring for patients with dementia and that communication is a particular problem [8, 9].

### Problems in dementia communication

Advice is available on how best to communicate with people with dementia, often in the form of ‘10 top tips’ (see for example, [10–12]). Not all recommended strategies are based on empirical research, however [13], and those that are evidence-based rarely originate from research in the general hospital. There is also evidence that strategies (such as speaking slowly) once believed to be effective do not reduce communication breakdowns, or can worsen the situation (27). This suggests that seeking expert (carer/patient/professional) opinion may not be adequate for planning training, and evidence-based approaches are required. A systematic review of dementia-specific communication skills training [14] identified no research on communication skills training in acute hospitals, nor any related to the training of doctors. We updated this systematic review [15] and found no approach which could be used or adapted directly with a mixed group of HCPs in the acute hospital setting.

### Conversation analysis of video data

Conversation analysis is a well-established qualitative method for the analysis of video-recorded social interaction [16–18] which has been used to develop successful communication skills training interventions in healthcare fields such as stroke [19], psychosis [20, 21] and primary care [22]. We applied conversation analysis to video recordings of experienced HCPs communicating in the acute hospital with PLwD, to identify the structure of encounters, the problems that arose, and what communication behaviours were more or less interactionally successful (VideOing to Improve Communication through Education', VOICE study). The study analysis focussed on two common and interactionally problematic areas for HCPs: making a request and responding to refusals [23]; and frequently-prolonged closings of healthcare encounters [7].

### Training development process

We used evidence from this analysis, together with a systematic review of dementia communication skills training [14, 15] and expert opinion, to develop a dementia communication skills training course for HCPs working with PLwD in the acute hospital. The training course was developed over a series of four whole-day intervention development meetings attended by: the researchers; three family carers of people living with dementia; educational experts; conversation analysts; experts at working with simulated patients in education; and clinicians expert at caring for PLwD. A pilot of the course with five HCPs with an interest in education allowed practice of the course in real time (‘dress rehearsal’). Feedback from these insightful trainees resulted in further refinement of the intervention.

## Methods

We undertook a before-and-after study of HCPs attending a two-day dementia communication skills training course. The purpose was to evaluate the training intervention, by measuring outcomes aligned to Kirkpatrick’s educational evaluation framework [24], namely confidence, knowledge, and acceptability of the course, and ratings of video-recorded simulated patient encounters.

### Training course intervention

We used experiential learning theory [25] to focus on learning in action, consequently much of the course required active participation by the HCP through simulated exercises. These involved actors, experienced in playing patient roles in healthcare education, who were trained as simulators of PLwD (known as simulated patients, or SPs). Characteristics of each actor’s role were specified by the research team, in collaboration with an experienced simulator, and based on real interactions analysed in the conversation analysis [26]. The preparation for SPs included watching a documentary of person-centred dementia care filmed on a ward, *Today is Monday* [27], watching films portraying people with dementia (such as *Still Alice [28])* and completion of three computer-based learning modules (reusable learning objects, RLOs; these are 15–20 minutes of focused multimedia and interactive online teaching and learning, on dementia, person-centred care and basic communication skills) [29–31]. A one-day taught course for the SPs included a geriatrician-led discussion on how someone with dementia and communication impairment might present, the showing of video-recorded material from the conversation analysis study, and an opportunity to practice the SP role with members of the study team (a nurse, and speech and language therapist, SLT).

### Training delivery

The course comprised two days, one month apart. Participants were asked to prepare by completing computer-based learning modules if they felt they needed to. The first day included the typical structure of a HCP-initiated interaction, an introduction to the different forms of request and possible responses, the making of a request of the patient to participate in or co-operate with a task, managing subsequent acceptance or refusal and closing an interaction. We used edited video data from the conversation analysis study to support learning (having gained prior written consent to use videos for this purpose).

Participants then took part in small-group, facilitated simulation workshops. Each participant was asked to role-play a scenario with an SP in turn. A range of tasks was offered, for example, doing a gait or swallow assessment, or supporting the patient to wash their face, enabling each participant to choose a task appropriate to their profession. The SPs had been briefed to initially refuse to carry out the tasks when requested and to prolong the closings. While each participant performed the scenario, the other 3–4 participants and the facilitator observed, and prepared structured feedback on the communication encounter. Out of role, the SP gave feedback on how they felt during the interaction. Participants were instructed to be supportive and constructive in their feedback. There was opportunity to restart or replay the interaction, ‘rewind’ to an earlier point, or to take ‘time-out’ and ask for the advice of the group.

Participants were asked to complete a reflective diary over the month between the training days including one example where they successfully used the communication techniques taught and another which was less successful. These diaries were used as part of a reflective workshop on the second day. Participants were asked to complete a further RLO, developed to support the course, to reinforce learning.

The second day included small–group workshops using the reflective diaries, a session on person-centred care [32] and avoiding ‘elder speak’ [33] (infantilising talk aimed at older people), a video workshop to reinforce teaching from the two days, and a second simulation workshop involving more interactionally challenging simulations of reluctance/refusal and delayed closings.

The courses took place in dedicated clinical skills teaching centres in two hospitals in the UK.

### Participants

Participants were volunteers, approached via posters, word-of-mouth or their line manager. They were registered HCPs including doctors (consultants, registrars and core medical trainees), occupational therapists, physiotherapists, speech and language therapists, nurses, an orthotist, and an activities co-ordinator. All worked regularly with PLwD. A clinical researcher (ROB, a registered SLT) discussed the study with potential participants and if they met inclusion criteria they were sent a participant information sheet and consent form. Written informed consent was taken on the morning of the first day of training.

### Measures

The knowledge and confidence of participants before and after training, and their views on acceptability of the course were measured by self-completed questionnaire. Changes in communication behaviour were measured by blind rating of simulated assessments. Follow-up questions on recall and practical use of the skills were asked by email one month after the course (Table 1).

**Table 1:** Timing of when each outcome measure was taken.

Measure	Baseline	End of day two of course	One month after day two of course.
Demographics	√		
Confidence in dementia	√	√	
Dementia Communication Knowledge Test	√	√	
Question on awareness of communication skills,	√	√	
Question on use of communication skills,	√	√	
Confidence ending a conversation where the patient tries to continue it, achieving a task in the person with dementia’s best interest when their first response is a refusal, awareness of the best way to ask someone with dementia to do something.	√	√	
Confidence at achieving a task in the person with dementia’s best interest when their first response is a refusal,	√	√	
awareness of the best way to ask someone with dementia to do something	√	√	
Evaluation of the training questionnaire		√	
Questions on: Do you remember the skills learnt on the training course Are you performing the skills learnt Do you consider the skills useful in your role as HCP.		√	√
Simulated assessment	√	√	

#### Questionnaires

At baseline participants completed:

- Demographic information.
- The Confidence in Dementia Scale (‘CODE’) [34]. This is a nine-item scale to assess a person’s confidence at caring for a PLwD, for example ‘I feel able to manage situations when a person with dementia becomes agitated’. A Likert scale for responses from 1 (not able) to 5 (very able) was completed for each item. Three additional questions linked to the skills taught on the course were developed by the research team (how to make a request, communicating following a refusal and how to close a healthcare conversation). These asked participants to rate their confidence on a scale of 0 (no confidence) to 10 (totally confident). The CODE has been shown to have good internal consistency, low item redundancy and very good sample adequacy (Cronbach’s alpha of 0.88; overall Kaiser-Meyer- Olkin 0.89) [34, 35]
- Dementia Knowledge Test–this was developed by the research team to test knowledge of communication with PLwD. It comprised 10 items with multiple-choice answers (S1 Table).

At the end of the second day of training, participants were asked to complete the following questionnaires:

- Confidence in Dementia Scale
- Five questions, developed by the research team asking participants to rate their confidence on a 0–10 scale (0 being no confidence and 10 being total confidence) on awareness of communication skills, use of communication skills, ending a conversation where the patient tries to continue it, achieving a task in the person with dementia’s best interest when their first response is a refusal, awareness of the best way to ask someone with dementia to do something.
- Dementia Communication Knowledge Test
- Evaluation of the training course. Participants were asked to rate on a scale of 1 to 10 whether the course was interesting, useful, informative, and enjoyable, did they feel respected, and safe, was the course challenging and relevant to their practice, did the course fulfil their learning goals, and improve their practice. They were also asked if the course met their expectations and if they would recommend it to their colleagues.

At the end of the second of the course, and one month later, participants were asked if: they remembered the skills they had learnt; were using the skills; and if they considered the skills to be useful in their work.

Participants were asked to record free text comments on the course about what they had learnt; what was most helpful; how would it help with caring for PLwD; any suggested changes; and any further comments.

#### Simulated encounter measure

Participants undertook a video-recorded simulated exercise before and after training. They were given one of two scenarios, containing brief details about the ‘patient’ and the generic healthcare task to be completed, which was either to get the simulated patient out of bed, or get the patient to drink some water and eat a biscuit. They were asked to close the interaction and leave the room as if they were dealing with a real patient. The SPs enacting these assessment scenarios did not perform in the simulation workshops during the same training course. SPs were asked to refuse the task several times and to extend the closing of the interaction. If the encounter was continuing after 10 minutes had elapsed, an indicator was given (a knock at the door) to prompt the HCP to close the encounter and leave as soon as was appropriate. Each participant completed the assessment with a different role at baseline and outcome; half the group did the baseline assessment with one role and the other half with the other role, in a cross-over design.

A checklist was developed to assess communication behaviours in the video-recordings (S2 and S3 Tables). This checklist reflected the content of the training course, and identified specific objectively-identifiable communication behaviours, which had been identified in the conversation analysis and taught on the training course. Ratings were made independently by two trained, experienced SLTs, blind to whether the interaction was before or after training. Videos were edited visually (blurring clocks) and auditorily (silencing greetings which included morning/afternoon) to remove references to time of day which might have unblinded the raters. Video data from the pilot course were used to practice and reach acceptable levels of agreement between raters. A random number generator was used to assign videos in a random order. Ratings are reported where both raters agreed.

Video recordings were also rated by six Public and Patient Involvement (PPI) representatives, in order to check whether patients and families would consider any changes in HCPs’ communication behaviours ‘acceptable’- that is, that HCPs would appear no less person-centred after the training than before. All the PPI raters either had dementia themselves or had experience of caring for PLwD and were recruited via the University of Nottingham’s dementia and frail older person PPI group or the Alzheimer’s Society PPI monitoring group. The PPI representatives used the Emotional Tone Rating Scale which is a valid and reliable scale designed to ‘measure the underlying affective qualities of communication with older adults’ [36]. According to the authors, minimal training of users is needed. It includes 12 items chosen to capture the emotional tone of communications, rated using a five point Likert scale (1 = not at all, to 5 = very). We asked raters to assess: ‘The healthcare professional’s communication was…nurturing, directive, affirming, respectful, patronising, supportive, polite, bossy, caring, dominating, warm, controlling’. The PPI raters were not informed that the videos were before and after a training course, and videos were presented in random order. Encounters were rated after watching two minutes of video: one minute starting from the HCP’s first request, and one minute taken from the start of the closing sequence. The time points were those previously documented by the SLT raters. The two minutes of video recording were each played twice. Two PPI representatives rated 42 encounters; one rated 22 encounters, one rated 23 encounters, two rated 20 encounters.

### Sample size

45 participants were recruited to represent a range of individuals and healthcare professionals, whom it would be feasible to train over a six month period. Other studies using a before-and-after design to evaluate dementia communication skills training used sample sizes ranging from 15 to 48 [5, 37–41].

### Statistics

Data were summarised using descriptive statistics. Differences in responses before and after training were assessed using paired t-test and the Wilcoxon signed-rank with 95% confidence intervals.

Kappa statistics were calculated for the SLT and PPI ratings of the simulation assessment video recordings to test inter-rater agreement. Changes in the Emotional Tone Rating Scale were assessed using paired t-test.

McNemar’s test was used to assess whether there was a change in the taught communication behaviours before and after the training. The McNemar exact test was used when the discordant pairs totalled <20.

## Results

Between January and May 2017, 45 healthcare professionals attended one of six VOICE training courses, with each course attended by six to nine participants. The healthcare professionals comprised 8 (18%) doctors; 19 (42%) nurses, 17 (38%) allied health professionals and one activity co-ordinator. Eighty-nine percent were female. Participant ethnicity was 89% white, 9% Asian and 2% mixed. The median number of years of experience working with patients with dementia was five (Table 2). Twenty-nine (64%) participants attended training at site one. 44/45 participants attended both days of the training course. Baseline questionnaires for one participant were not returned, despite repeated requests. The analysis of self-reported scales is therefore confined to 43 participants.

**Table 2:** Demographic characteristics of participants (n = 45).

	Frequency (%) N = 45	Median (Range) (Years)
Profession:		
Doctors	8 (18%)	-
Nurses	19 (42%)	-
AHPs	17 (38%)	-
Activities co-ordinator	1 (2%)	-
Years of experience working with patients with dementia (IQR)	-	5 (3–8)
Gender:		
Female	40 (89%)	-
Male	5 (11%)	-
Ethnicity		
White	40 (89%)	-
Asian	4 (9%)	-
Mixed	1 (2%)	-

### Confidence in dementia

Confidence in dementia improved following the course in all categories, both on the Confidence in Dementia Scale (32.8/45 before and 38.3/45 immediately after the course). Mean improvement in total Confidence in Dementia score was 5.5 (95% CI 4.1–6.9). Improvement on the dementia knowledge test from baseline to immediately after training was on average 1.5/10 points (95% CI 1.0–2.0) (Table 3).

**Table 3:** Results of outcome questionnaires.

Outcome measure	Pre score Mean N = 43	Pre score mean 95%CI	Post Score Mean N = 43	Post score mean 95%CI	Difference N = 43	Difference 95%CI	P value
Confidence in Dementia Scale *(scored on a Likert scale of 1 (not able) to 5 (very able))*	32.8	31.6–34.1	38.3	37.2–39.5	5.5	6.9–4.1	<0.001
Confidence in ending a conversation where the patient tries to continue it *(scale 0 to 10 where 0 is no confidence and 10 is totally confident)*	4.5	3.7–5.3	7.8	4–10	3.3	2.3–4.3	<0.001
Confidence in achieve a task in the persons best interest when there first response is a refusal *(scale 0 to 10 where 0 is no confidence and 10 is totally confident)*	4.5	3.8–5.3	8.2	6–10	3.7	2.8–4.5	<0.001
Awareness of best way to ask someone with dementia to do something*(scale 0 to 10 where 0 is no confidence and 10 is otally confident)*	4.7	3.9–5.4	8.7	6–10	4.0	3.1–4.9	<0.001
Dementia Communication Knowledge Test *(10 questions*, *correct answers scored 1; incorrect answers scored 0)*	7.2	6.8–7.7	8.8	8.4–9.1	1.5	1.0–2.0	<0.001

### Acceptability and satisfaction

Almost all participants (95%) found the course met their expectations and 98% would recommend the course to other HCPs. The course evaluated highly in other respects, with mean scores of 9/10 or higher on statements on whether the course was interesting, useful, informative, enjoyable, relevant to their practice, fulfilled their learning goals and improved their confidence, and on whether they felt respected and safe. The statement ‘The course was challenging’ had a mean score of 8.4/10 (Table 4).

**Table 4:** Course evaluation (scored on a scale of 1 to 10, 10 affirming the statement).

Question	Mean score out of 10 (range) N = 44
Do you remember the skills you learned I the training course	8.7 (6–10)
Are you performing the skills you learned in the training course?	8.2 (6–10)
Are the skills helpful in your role as a healthcare professional?	9.6 (8–10)
**The course was:**	
Interesting	9.3 (7–10)
Useful	9.4 (7–10)
Informative	9.4 (7–10)
Enjoyable	9.1 (7–10)
I felt respected	9.7 (8–10)
I felt safe	9.8 (7–10)
Challenging	8.4 (3–10)
Relevant to my practice	9.5 (7–10)
Fulfilled my learning goals	9.1 (5–10)
Improved my confidence	9.2 (6–10)
**Confidence in**	
Awareness of communication skills	8.6 (7–10)
Use of communication skills	8.5 (7–10)

High ratings were given to the questions asking participants if they remembered the skills (8.7/10); if they were performing the skills (8.2/10) and whether the skills were helpful (9.6/10).

### Written feedback

The most valued parts of the course were: the simulation workshops including the immediate feedback provided and being able to practice the skills (mentioned by 27 participants); the specific techniques/skills learnt (mentioned by 8 participants) and the reflective exercise between the two days (mentioned by 5 participants). Being able to watch others undertake communication tasks, and interdisciplinary learning were also valued.

### Follow-up findings

The response rate one month after the second day of the course was 31/44 (70%). Participants gave a mean score of 8.6/10 to the question “do you remember the skills you learned in the training course?”; 8.4/10 for the question “are you performing the skills you have learned in the training course?” and 9.3/10 for the question “are these skills helpful in your role as a healthcare professional?”

### Communication behaviours

Agreement between raters (kappa) for each communication behaviour was fair or moderate. Following training, when closing an interaction, participants were less likely to make a vague arrangement (56% before versus 16% after; p<0.001); were more likely to be specific about closing the conversation (51% versus 79%; p = 0.01); and were more likely to announce completion of the task (0% versus 14%, p = 0.03). (Table 5).

**Table 5:** Blind ratings of communication behaviours during closings of evaluation simulations.

	Communication technique seen before training	Communication technique seen after training	McNemar’s test Odds Ratio (95% CI), p-value
Vague arrangement making	24/43 (56%)	7/43 (16%)	0 (0, 0.24); p<0.001
Specific closings	22/43 (51%)	34/43 (79%)	4 (1.3, 16.4); p = 0.01
Notification ahead of closing	7/43 (16%)	11/43 (26%)	2 (0.5, 9.1); p = 0.4
Announcing completion of task	0/43 (0%)	6/43 (14%)	n/a; p = 0.03
Announcing explicit intention to leave.	22/43 (51%)	23/43 (53%)	1.1 (0.42, 2.9); p = 0.8
Nonverbal actions supporting verbal actions	6/43 (14%)	6/43 (14%)	1 (0.2, 4.3); p = 1.0
Closing idiom used	16/43 (37%)	22/43 (51%)	2 (0.7, 6.5); p = 0.24
Anything else question asked	7/43 (16%)	4/43 (9%)	0.6 (0.1, 2.2); p = 0.55
Mismatch between verbal and non-verbal communication	1/43 (2%)	3/43 (7%)	3 (0.24, 158); p = 0.62

There were no significant changes in communication behaviours related to requests (Table 6). Some behaviour appeared resistant to change (for example both before and after training, 86% of participants did not make the initial request explicit; 79% did not make the subsequent request explicit; 95% did not soften the initial request by saying such things as ‘…is that okay?’). In addition, participants already used some of the taught requesting techniques prior to training (for example, 74% of healthcare professionals used language which conveyed their authority to make the request (entitlement) when making a follow-on request; 93% of healthcare professionals made the task seem smaller or easier (reducing contingencies) for follow-on requests).

**Table 6:** Blind ratings of communication behaviours during requests in evaluation simulation.

	Communication technique seen before training	Communication technique seen after training	McNemar’s test Odds ratio (95% CI); p-value
Initial request made in a highly entitled way	2/43 (4%)	8/43 (18%)	7.0 (0.9, 315); p = 0.07
Subsequent request made in a highly entitled way	32/43 (74%)	37/43 (86%)	2.2 (0.6, 10); p = 0.27
Initial request softened	2/43 (5%)	3/43 (7%)	1.5 (0.17, 18.0); p = 1.0
Subsequent request softened	8/43 (19%)	11/43 (26%)	1.4 (0.5, 3.9); p = 0.65
Initial request includes a reduction of contingencies	13/43 (30%)	9/43 (21%)	0.6 (0.2, 1.8); p = 0.45
Subsequent requests include reduction of contingencies	42/43 (98%)	40/43 (93%)	0.3 (0. 4.2); p = 0.62
Initial request is explicit	3/43 (7%)	3/43 (7%)	1 (0.1, 7.5); p = 1.0
Subsequent requests are explicit	2/43 (5%)	8/43 (19%)	7 (0.9, 315); p = 0.07

The PPI raters showed poor inter-rater reliability (kappa 0.013 to 0.097), but we report the results for completeness. On the Emotional Tone Rating Scale (with 1 indicating ‘not at all’ and 5 indicating ‘very’), the communication of the healthcare professionals was more controlling after the intervention, (2.2/5 versus 2.8/5; p = 0.002), bossy (1.9/5 versus 2.3/5; p = 0.02) and dominating (1.9/5 versus 2.5/5; p = 0.006). There was no difference in the other categories of communication tone (nurturing, directive, affirming, respectful, patronizing, supportive, polite, caring and warm).

## Discussion

We have created an innovative, evidence-based, dementia communication skills training course which is founded on experiential learning theory [25]. In doing so we have sought to enhance the authenticity of simulated interaction used in education and training. The course improved knowledge and confidence in communicating with PLwD in the acute hospital was acceptable to participants and changed aspects of communication behaviour. However, PPI raters observed an increase in controlling, bossy and dominating communication from HCPs after training. HCPs attending the course reported that one month after completing the course they still remembered, used and valued the skills they learned.

This is the first theoretically-informed dementia communications skills training course to be evaluated in the acute hospital setting. Evaluation of the course followed the first three of Kirkpatrick’s four level training evaluation model [24]. It measured i) reaction (we assessed whether the learning was a valuable experience), ii) learning (we assessed whether the participants’ knowledge increased after the course) and iii) behaviour (we assessed how the trainees applied the new information to their communication behaviours/practices in simulated assessments).

A limitation is that we were unable to formally measure whether the course changed patient outcomes, but we did ask whether the HCPs were using the knowledge and skills one month following the course. It is possible that the participants evaluated the course more favourably or reported greater post-course confidence to support the researchers, a social desirability bias. However, the video-recorded simulated interactions were rated ‘blind’ both by SLTs and PPI representatives. We chose to measure outcomes at one month after day one of the course to balance the need for a consolidation period with the practical challenges of achieving good response rates from staff who can regularly rotate wards. And in fact the response rate in the short time from the end of the course to one month later dropped from 98% to 70%. However, we acknowledge that the process of experiential learning requires time for a learner to assimilate new knowledge and skills. One month may have been insufficient time for participants to be able to demonstrate all the new communication skills learnt on the course; this may be one factor to account for a lack of change in requesting behaviours.

The course was developed by a multidisciplinary team, including three carers of people with dementia. While PLwD were not involved in the intervention development as recently recommended by Alzheimer’s Europe [42], PLwD were involved in the PPI rating of the before and after video simulation assessments.

The SLT ratings of the simulated encounters showed a change in behaviours around one training theme, communication to close the encounter, but not for the other, requesting. For some communication behaviours (such as lowering contingencies on follow-on requests), this was because the participants were already demonstrating the skills prior to the course. However, an important role for training is to educate HCPs in what they already do well. This can result in the HCPs being more confident in their competence [43] and gives them a language to articulate what they do well to members of staff they are managing or mentoring. Measuring changes in complex communication behaviours is challenging. We used a checklist of communication behaviours taught on the course and the before and after simulation assessments were rated by experienced SLTs trained in the checklist and the relevant communication behaviours. However, reducing complex communication behaviours to decontextualised checklist items may not have been sufficient to measure changes effectively.

The PPI raters using the emotional tone rating scale identified an increase in ‘dominating’, ‘controlling’ and ‘bossy’ communications after the training. Such effects would be contrary to the values of compassionate care. We emphasised the importance of person-centred care throughout the training, however this aspect should be kept in mind in future development of the course. We should emphasise that ambiguous or unnecessarily complicated language may sound polite, but be ineffective and potentially distressing if not understood. By contrast direct requests may be perceived as ‘controlling’ and at the same time increase the likelihood of successful completion of important healthcare task with PLwD.

Since the English National Dementia Strategy (2009) [44], there has been a focus on training HCPs in how to deliver better healthcare to PLwD. NICE guidelines on dementia care emphasise that: “Health and social care managers should ensure that all staff working with older people in the health, social care and voluntary sectors have access to dementia-care training (skill development) that is consistent with their roles and responsibilities. (NICE 2016, 1.1.9.1 [45])”. HCPs in hospital are often expected to perform tasks with PLwD that require co-operation and to complete tasks within a limited amount of time. Strategies that increase HCP efficiency without disadvantaging the patient should be beneficial. While the advantages to the hospital systems are clear, more research may be needed to verify that the impact on patients is not detrimental.

There is some debate about whether simulation is an effective way to assess and train communication skills, including with HCPs, with research evidence to suggest that simulated patients can be inauthentic and do not respond in the same way as real patients [46–48]. Others have found the use of simulated patients in training to improve nurse’ knowledge and attitudes towards illness and death [49]. Students themselves have reported that simulation gives them the opportunities to practice ‘difficult’ encounters such as breaking bad news, and dealing with angry patients [50], though interactions with real patients were considered to be more motivating in terms of researching particular health conditions.

In the VOICE training course, we aimed for the simulated patients to be ‘not inauthentic’ by training simulators using research findings and by basing their scenarios on real recorded encounters. The VOICE training included both use of real video and simulated encounters. We believe there are benefits to practicing a communication skill in real time within a safe and supportive environment where others can comment on and learn from another’s performance. Simulation gives participants the opportunity to observe how others communicate. The simulation was frequently commented on by participants as one of the most helpful aspects of the course. The reflective exercise between the two days of the course gave participants the opportunity to practice their communication skills on real patients, and reflect, in a supported way, on their abilities and the consequential outcomes.

The course was developed with considerable service-user involvement, and the two-day duration of the course and the inclusion of a reflective exercise between the two days were both components which were championed by service-users. The educators and healthcare professionals developing the training thought the content justified two days. A shorter course could only have considered more isolated aspects of interaction, rather than focusing on recurring interactional issues in the context in which they actually occur. Many of the HCP participants were senior members of staff and will be able to influence others by role modelling and bedside education, thus extending the reach of this training beyond those who have directly attended.

This research was based on research findings from natural, real life encounters about what works; the intervention was developed by a multi-disciplinary team including pedagogical experts. Delivery of the course was through experienced healthcare educators, with expert SPs. Our evaluation was rigorous and used an established framework [24]. To assess changes in behaviour, we innovatively used blind-rated simulated encounters to assess changes in communication skills and behaviours.

However, the participants were not randomly selected; we trained volunteer HCPs who were interested in dementia and improving their dementia communication skills, and many of whom already had well-developed skills. The research used a before and after design, which is prone to bias. There may have been a social desirability bias when completing the scales. Whilst the CODE confidence in dementia scale has robust psychometric properties, the Dementia Communication Knowledge Test and the evaluation of training were developed by the research team in the absence of suitable published tools, and we have not tested the psychometric properties of these questionnaires.

We have trained few HCPs who have English as a second language. Given the demographics of the UK National Health Service, further research is required on the communication patterns of HCPs where English is not the first language. We focused our research on the acute hospital. More work is needed on developing dementia communication skills training courses based on empirical evidence of what actually happens in practice in other settings such as care homes and patients’ own homes.

## Conclusion

All HCPs who care for patients with dementia need dementia-specific communication skills training. The course we developed could provide such training. However, a two-day course may be considered time-intensive and the use of simulated patients has an expense associated with it. We argue that delivering training effectively (that is, training which produces lasting behaviour change of benefit to staff and patients in the NHS) requires an investment.

## Supporting information

S1 Table Dementia communication knowledge questionnaire. (PDF)

S2 Table Interrater reliability of SLT-blind ratings of the presence or absence of communication behaviours in making requests during evaluation simulation. (PDF)

S3 Table Interrater reliability of SLT-blind ratings of the presence or absence of communication behaviours in closings during evaluation simulation. (PDF)
