# Supplementary material for: The VOICE study – A before and after study of a dementia communication skills training course
Source: PLoS One. 2018 Jun 11;13(6):e0198567. doi: 10.1371/journal.pone.0198567 (PMC5995402; doi:10.1371/journal.pone.0198567)
Supplement: S1 Table — (PDF) [file pone.0198567.s001.pdf]

S1 Table. **Dementia Communication Knowledge Questionnaire**

No.	Question
1	When communicating with people with dementia it's best to speak:<br>a) Fast and clearly<br>b) Slowly and clearly<br>c) At a normal rate and clearly
2	When approaching a patient with dementia to carry out a healthcare task the best introduction would be:<br>a) Hello Margaret. Do you remember me?<br>b) Hello Margaret. I'm Diane, one of the doctors here. I've come to see if you're getting better.<br>c) Hello Margaret. Can I check your blood pressure?
3	Which of these communication strategies might help when communicating with someone with dementia:<br><br>A). Using gestures, objects or pictures to show what you mean<br><br>B). Using metaphors to explain things.<br><br>C). Touching the part of the body you are talking about.<br><br>D). Using short sentences<br><br>E). Using one step instructions<br><br>a) A, B, C, D, E<br><br>b) A, C, D, E<br><br>c) A, B, D, E
4	If a patient with dementia is distracted, what is the best way to get their attention so you can talk with them?<br>a) Use their name<br>b) Speak loudly<br>c) Ask the relative rather than the patient.
5	Repeating back what you understand of what a patient just said to you, when you don't completely understand them, is likely to be:<br>a) A useful way of indicating you are listening and trying to understand.<br>b) Confusing for someone with dementia<br>c) Annoying for someone with dementia.
6	When requesting a particular patient with dementia takes an important medication, which you know they are often reluctant to do, it may help to:<br><br>a) Frame the request as a question about their willingness to do it, such as 'Joan, do you want to take your tablet now?'<br><br>b) Frame the request as a very polite question, such as 'Joan, I was wondering if you might possibly want to take your tablets now?'

	c) Frame the request as a statement of what you are proposing will happen, with a checking question at the end, such as 'Joan, I've brought your tablets for you to take now. Is that okay?'
7	When a patients says or communicates 'no' to doing something you have asked (and which the team and family thinks is important and in their best interest), which of the following approaches would be <u>unhelpful?</u><br><br>a) Keep repeating the request in the same way, slowly and clearly, until they agree<br><br>b) Make the task sound less demanding, by reducing the size or duration of the task eg. 'just for a minute'<br><br>c) Say that you need them to do it eg. 'I need you to take these, for your diabetes'
8	Towards the end of your session, if you ask the patient an open question like 'Is there anything else you want to ask me?' this is likely to lead to the patient with dementia:<br><br>a) being silent<br><br>b) being confused about what they are expected to say and not reporting any healthcare concerns<br><br>c) making some attempt to share their healthcare concerns or questions with you
9	To indicate to the patient that the session is about to finish, in a way that feels respectful, which of the following strategies/statements would work best?<br><br>a) I'll see you soon<br><br>b) I'll see you tomorrow morning<br><br>c) You're doing really well, and there's nothing to worry about
10	As you are ending a session with a patient on the ward, if you stand up, clear away your equipment and pull the curtains back, this is likely to:<br><br>a) appear rude to the patient with dementia<br><br>b) make no difference to the patient with dementia as they won't notice or understand these signals<br><br>c) help the patient with dementia understand that you are about to leave

4

5
