# Supplementary material for: The VOICE study – A before and after study of a dementia communication skills training course
Source: PLoS One. 2018 Jun 11;13(6):e0198567. doi: 10.1371/journal.pone.0198567 (PMC5995402; doi:10.1371/journal.pone.0198567)
Supplement: S2 Table — (PDF) [file pone.0198567.s002.pdf]

S2 Table. Interrater reliability of SLT-blind ratings of the presence or absence of communication behaviours in making requests during evaluation simulation

**Communication practice**	**Example**	**Inter-rater reliability first request (kappa)**	**Inter-rater reliability subsequent request (kappa)**
High entitlement request: proposal	Let's: (Let's try a yoghurt).	0.48 Moderate	0.69 Substantial
High entitlement request: announcing future action	Going to/we'll	0.22 Fair	0.57 Moderate
High entitlement request: statement of need	I need you to; I need to; You need to	0.59 Moderate	0.55 Moderate
High entitlement request: direct instruction	Take a step	0.32 Fair	0.39 Fair
High entitlement request softened eg. with checking / permission-seeking question	Is that okay? Alright? Okay?	0.43 Moderate	0.47 Moderate
High entitlement: Other	Forced alternatives which presumes compliance ('Which finger shall I use?')	0.42 Moderate	0.24 Fair
Lowering contingencies: Reduces the size or duration of task	Just, little, pop, quick, for a minute:	0.12 poor	0.55 Moderate
Lowering contingencies: Request includes 'try'	Try: (Shall we give it a try then?)	0.66 Substantial	0.64 Substantial
Lowering contingencies: Explicit offer to help	(What about if I give you a hand?)	0.31 Fair	0.79 Substantial
Lowering contingencies: Frame accurately as collaborative or joint action	We; let's; for me: (Shall we go for a walk);	0.49 Moderate	0.17 Slight
State the action explicitly (not just stating the reason for the action)	(What I want to do is give you a shave)	0.08 poor	-0.02 poor
Action required of patient is not stated explicitly	(Can I take your blood pressure?)	n/a	n/a
