# Supplementary material for: The VOICE study – A before and after study of a dementia communication skills training course
Source: PLoS One. 2018 Jun 11;13(6):e0198567. doi: 10.1371/journal.pone.0198567 (PMC5995402; doi:10.1371/journal.pone.0198567)
Supplement: S3 Table — (PDF) [file pone.0198567.s003.pdf]

**S3 Table. Interrater reliability of SLT-blind ratings of the presence or absence of communication behaviours in closings during evaluation simulation**

| Communication practice during closing | Examples | Reliability (kappa) |
|---|---|---|
| Vague arrangement at closing | (See you soon; See you around) | 0.51 Moderate |
| Specific closing arrangement | (See you tomorrow; I'll get that cup of tea now.) | 0.25 Fair |
| Notification ahead of final activity | (Before I go… ) | 0.31 Fair |
| Announcing completion of final activity | (That's us all done). | 0.42 Moderate |
| Announcing explicit intention to leave | So I'm gonna go now. | 0.31 Fair |
| Non-verbal actions supporting verbal closing | (Re-positioning table; tidying equipment) | 0.40 Fair |
| Closing idiom or saying | (All done and dusted; I'll leave you be; We'll keep a close eye on things; You take care). | 0.29 Fair |
| 'Is there anything else?' type open question during closing | (Anything you want to ask me before I go? Is there anything I can help with?) | 0.37 Fair |
| Mismatch between nonverbal and verbal actions during closing | E.g. HCP gives verbal indications of closing but doesn't make physical moves to indicate closing/leaving; HCP opens new lines of enquiry (verbal) whilst walking away (non-verbal). | 0.41 Moderate |
